# Climate Justice Perspectives and Experiences of Nurses and Their Community Partners

**DOI:** 10.1111/nin.12690

**Published:** 2024-12-16

**Authors:** Jessica LeClair, Alex Dudek, Susan Zahner

**Affiliations:** ^1^ School of Nursing University of Wisconsin—Madison Madison Wisconsin USA; ^2^ School of Medicine and Public Health University of Wisconsin—Madison Madison Wisconsin USA

**Keywords:** climate justice, community‐based organization, nursing, participatory photo mapping, partnership, photovoice, planetary health, public health

## Abstract

The global climate crisis is an immediate threat, causing inequitable health impacts across different populations. Climate justice connects the causes and effects of climate change to structural injustices in society. Nurses and community‐based organizations (CBOs) partner in promoting justice and health equity. The purpose of this article is to describe how nurses and their CBO partners envision, perceive, and experience climate justice in the communities they serve. Participants were recruited via a screening survey sent to nursing and public health organizations in the United States. This descriptive mixed‐methods study utilized participatory photo mapping (i.e., combined participatory photography, community mapping, and interviews) to capture participants’ understanding and experiences of climate justice. Recruitment methods identified eight partnerships across six states. Participants depicted how climate injustice is reinforced by colonial severance from Nature. Participants noted that state violence and corporate climate pollution degraded the public's health. Climate justice was described as a long struggle to regain spiritual relationships within Nature, fostering belonging, abundance, and protected communities of care. Planetary health and well‐being were central to participants’ experiences with climate justice. Future research could explore barriers and facilitators to addressing climate injustice and promoting climate justice in diverse settings.

## 1 Introduction

The global climate crisis is a public health threat (World Health Organization [WHO] 2021). Evidence of worsening morbidity and mortality in the United States is associated with climate change (Hayden et al. 2023). Public health impacts include the direct effects of heat waves, flooding, and changes in the frequency and intensity of other extreme weather events (Bekkar et al. 2020; Ebi et al. 2021). Public health threats from climate change also include injuries and premature deaths related to extreme weather events. For example, since 1980, the United States has experienced an estimated 14,223 deaths from extreme weather and climate disasters, although this number is likely an underestimate (Ebi et al. 2021). Researchers have also identified increased numbers and kinds of infectious diseases and increased mental illness among diverse populations across the United States (Ebi et al. 2021; Hayden et al. 2023; Landrigan et al. 2024).

There are inequitable climate‐related health impacts across different populations (Benz and Burney 2021; Hayden et al. 2023). People with vulnerable physiology, such as children, pregnant people, and older adults, have disparate health outcomes because of the climate crisis (Bekkar et al. 2020; Hayden et al. 2023). Centuries of colonial domination (i.e., political, economic, social, and cultural control over colonized peoples) guarantee disparate and inequitable climate impacts among Black and Indigenous communities due to the extraction of resources and labor for economic gains (Bullard 1994; Redvers et al. 2022). These populations, as well as other racialized groups and low‐income communities, are often on the “frontlines” of climate disasters and along “fencelines” to industrial pollution (Baptista et al. 2023; Bullard 1994; Garibay and Arevalo 2017; Rockström et al. 2023). Frontline communities experience climate change impacts first and worst, such as when homes lack the infrastructure to withstand heat emergencies or other climate disasters (Baptista et al. 2023; Garibay and Arevalo 2017; Rockström et al. 2023). Bens and Burney (2021) found that census tracts in poor neighborhoods and neighborhoods with higher percentages of Black, Latine, and Asian/Pacific Islander populations were overburdened with heat compared to wealthier, whiter neighborhoods. Fenceline communities live close enough to a toxic or industrial environment to experience direct harm from the associated pollution (Bullard 1994). For example, 14.1 million residents who identify as Black, Latine, or Asian/Pacific Islander live in United States counties with the worst air pollution (Grineski and Collins 2018).

Climate justice is a movement that connects the causes and effects of climate change to structural injustices in society and recognizes that communities are entitled to equal protection by environmental and public health laws and regulations (Bullard 1990; International Climate Justice Network 2002). Climate justice scholars highlight that all climate justice action requires a transformative vision for justice and a relentless will to persist and survive through experiences of injustice (Battle 2020; Curley and Lister 2020; Sugla 2021; Whyte 2018, 2021). Nurse scientists increasingly seek to inspire the discipline to advance climate justice (ANA 2022, 2023; APHA 2013; LeClair, Evans‐Agnew, and Cook 2022; Lilienfeld et al. 2018; Nicholas and Breakey 2017; Travers et al. 2019). In support of these efforts, members of the Alliance of Nurses for Healthy Environments Global Climate Justice in Nursing Steering Committee (Climate Justice in Nursing Steering Committee 2021) utilized principles and frameworks from the climate justice movement to guide the development of an innovative Climate Justice in Nursing definition and the Global Nurse Agenda for Climate Justice (“CJ Agenda”) to help inform climate justice strategies in nursing research, education, advocacy, and practice (LeClair, Evans‐Agnew, and Cook 2022). The definition of climate justice in nursing is as follows:

Climate justice in nursing addresses the social, racial, economic, environmental, and multispecies justice issues of the climate crisis through centering the experiences and ways of knowing in frontline and fenceline communities and safeguarding the rights of Nature to achieve planetary health. (LeClair, Evans‐Agnew, and Cook 2022)

The CJ Agenda utilized the definition of climate justice in nursing and outlined 36 principles to help guide nursing actions that support the movement for climate justice (Evans‐Agnew, LeClair, and Sheppard 2024). The process of creating the CJ Agenda expanded international interest in nursing partnerships for climate justice and planetary health (Climate Justice in Nursing Steering Committee 2021; Evans‐Agnew, LeClair, and Sheppard 2024).

However, nurses consistently express a lack of preparedness to address the unjust health impacts of climate change in communities (Polivka, Chaudry, and Mac Crawford 2012; Zust and Jost 2022). Community‐based organizations (CBOs) working with frontline and fenceline communities have essential perspectives on approaching climate justice work. Because community residents drive CBOs, understanding CBO representatives' visions, perspectives, and experiences could inform nursing actions to advance climate justice (National Community‐Based Organization Network 2004; Simon‐Ortiz et al. 2024). While nurses and CBOs partner on various health issues, there remains a lack of published information on effective nurse and CBO partnerships to advance climate justice (ANA 2022; APHA 2013; Evans‐Agnew, LeClair, and Sheppard 2024; Kulbok et al. 2012; LeClair, Watts, and Zahner 2021; Zahner 2005). The purpose of this study was to describe how nurses and their CBO partners envision, perceive, and experience climate justice in frontline and fenceline communities.

## 2 Materials and Methods

**Figure 1:**
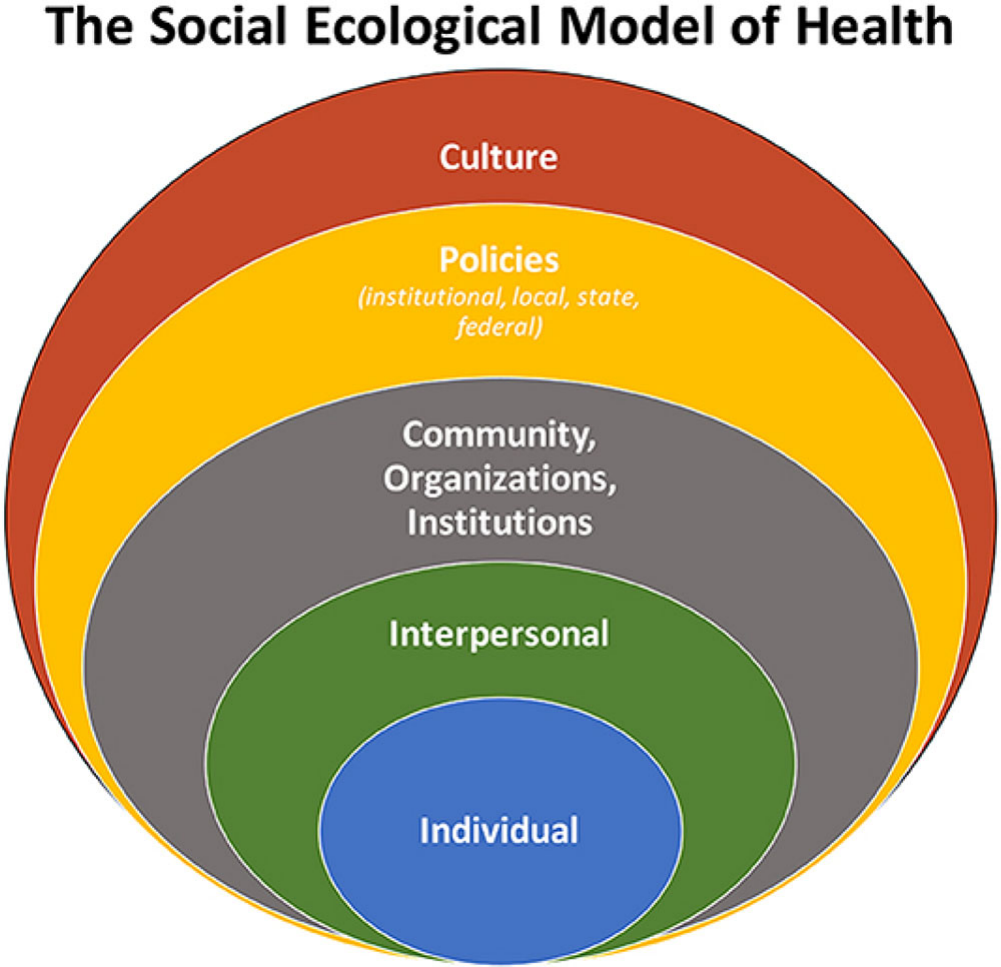
The social ecological model of health.

**Figure 2:**
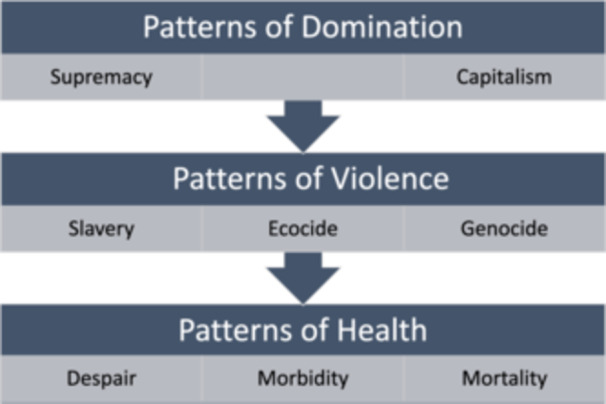
The critical environmental justice nursing for planetary health framework.

A descriptive, mixed‐method convergent research design was used. The University of Wisconsin‐Madison Minimal Risk Research IRB determined the study met the exempt criteria. The Social Ecological Model of Health was integrated to promote recognition of the social and structural determinants of health and health equity (Figure 1 by Golden and Wendel 2020, no additional adaptations were made, and it is shared under the Creative Commons Attribution 4.0 International Deed). Social Ecological Models have evolved, and the core tenants used in this project are designed around the interactive characteristics of individuals, interpersonal interactions, communities/organizations/institutions, policies, and the cultural environments that underlie public health outcomes (Golden and Wendel 2020). Human behavior can be observed and addressed at each of the levels. The Critical Environmental Justice Nursing for Planetary Health Framework (EJ Nursing Framework) was used to conceptualize the root causes of climate injustices as patterns of domination that degrade all life (Figure 2 by LeClair, Luebke, and Oakley 2021). These patterns of domination (e.g., human/male/white supremacy and capitalism) disadvantage many people, leading to despair, morbidity, and mortality (Bullard 1994; Kojola and Pellow 2021). Specifically, the framework applies Indigenous, Black, postcolonial, and eco‐feminist theories to exemplify how environmental injustices are often gendered, including exploitation of the Earth, and illustrate the consequences of the exploitation of colonized populations (LeClair, Luebke, and Oakley 2021).

### 2.1 Participant Recruitment

National recruitment was conducted by providing a study description and screening survey link to members of the Council of Public Health Nursing Organizations and state‐level nursing (*n* = 49 states and 2 territories), public health (*n* = 54), and climate and health organizations (*n* = 18) via their organization email lists, newsletters, or on their websites. Snowball recruiting was also used when some organization members shared the opportunity with nurses who were not members or sent the primary author contact information for the nurses they believed met the inclusion criteria for the study.

Criterion sampling was utilized to select participants (Polit and Beck 2017b). Interested nurses accessed the screening survey through the link provided. Nurses were invited into the study if they were (1) Registered Nurses, (2) engaged in the practice of promoting and protecting the health of populations (APHA 2013), and (3) self‐identified as having experience partnering with a CBO to advance climate justice in a frontline or fenceline community. Interested nurses shared the invitation with their CBO partners to assess the opportunity to participate.

CBO representatives were included in the study if they were (1) identified by nurses as community partners and (2) self‐identified as having experienced partnering with the nurse to advance climate justice in a frontline/fenceline community. If a CBO representative could not participate in the study, the nurse could enroll alone for partial data collection.

Cash incentives of $150 were offered to each nurse and CBO participant. Nurses and CBO participants confirmed their interest in study participation during a Zoom meeting with the primary author, during which signed consent was obtained, and individuals were enrolled.

#### 2.1.1 Sample

Fifty‐two nurses self‐screened as eligible to participate through the recruitment survey, and there were eight additional snowball referrals for potential nurse participants. Of these, 31 nurses did not respond to multiple outreach attempts. Twenty‐one nurses declined to enroll without providing a reason, cited lack of time given current work, or were ineligible due to lack of a suitable CBO partner or not being an RN during their experience working to support climate justice. The final sample was thirteen participants, which included eight nurses (13% of eligible participants) and five CBO representative partners. Three of the eight nurses participated without a CBO partner. Information about the reasons for doing the research was shared with all participants in the Research Information and Consent document. All participants could ask questions during the consent and enrollment process and at any time during their participation in the study.

Study participants were in Massachusetts, New Jersey, Wisconsin, Michigan, Ohio, and Washington. Nurses enrolled in the study reported working in academic or non‐profit settings. Participant demographic information was not collected. However, it was noted when participants self‐disclosed their social identities during the interviews. For example, several participants mentioned they identified with multiple intersecting identities, such as Indigenous, Black, white, woman, man, and members of the LGBTQ2+ community. All people who enrolled participated throughout the entire duration of the study.

### 2.2 Instruments and Procedures

Participatory photo mapping (PPM) methodology was utilized to study how people interpret, understand, and navigate their environments (Dennis et al. 2009). Data were gathered from August 2022 to February 2023 through PPM and semi‐structured, in‐depth, one‐on‐one interviews with nurses and CBO representatives. Participatory photo mapping data collection and analysis consisted of the following: (1) Participants used their smartphones to take pictures of their community, documenting what climate justice looks like and means to them; (2) photos became the objects of interviews in which individual and collective narratives are attached to particular images during a virtual meeting with each nurse and CBO pair, or each nurse who enrolled alone; (3) images were mapped as part of a community‐level Geographic Information System. Participants owned the photos and decided which ones to share with the researcher. The photography approach to PPM employed an adaptation of the photovoice method. This allows for photo contextualization by having participants prioritize photos for discussion and answer specific questions for each chosen photo (Wang and Burris 1997).

Semi‐structured interview guides were created by the first author of this project (LeClair 2023). They were utilized with each participant to understand their unique experience as a partner to support climate justice in their community, to ensure that specific topics were covered, and to allow participants to provide as many explanations as they wished (Polit and Beck 2017a). The first author conducted a 1‐h semi‐structured interview with each participant during a separate scheduled time from the photovoice interview. Initially, open‐ended, generative questions were used, such as, “Tell me about how you came to practice in the community” (Polit and Beck 2017a). In‐depth interview processes reflected the EJ Nursing Framework through a proactive inquiry into systems of injustice that lead to climate injustices (LeClair, Luebke, and Oakley 2021). The guide had two levels of questions: (1) main themes and (2) follow‐up questions (Kallio et al. 2016). The main themes covered the study's aim, and participants were encouraged to speak freely about their perceptions and experiences. An overall sense of the concepts, themes, and processes was obtained from the interviews (Braun and Clarke 2006, 2019). All PPM training and interviews were held virtually using the HIPAA‐compliant Zoom platform for convenience and cost. The PPM interviews and subsequent semi‐structured interviews were digitally recorded, downloaded with a password‐protected computer, transcribed verbatim via Zoom transcription by hired graduate students, and checked for accuracy by the research team. All transcripts were returned to participants for comments and/or corrections.

### 2.3 Data Analysis

**Figure 3:**
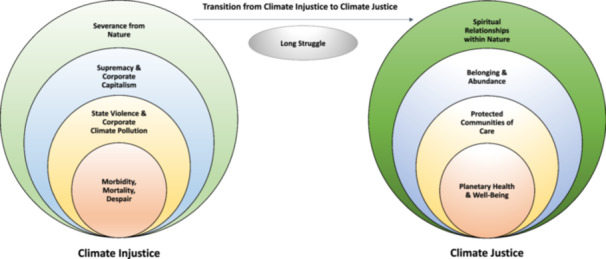
Climate justice perspectives and experiences of nurses and their community partners.

The constant comparative method guided the analytic approach, and data analysis began while data collection was ongoing (Boeije 2002). Using the photovoice method, participants critically analyzed what their photos depicted and what climate justice perspective they represented. These discussions facilitated the initial grouping of the data into substantive categories. As photovoice and individual in‐depth, semi‐structured interviews were conducted, cross‐group analysis was implemented of the initial themes identified by nurses and their community partners. The research team members independently listened to the recordings, read the transcripts, and wrote memos to facilitate analytic insights. Initial thematic codes were generated deductively from the previously described theoretical frameworks and then inductively from the data when information emerged for potential themes and subthemes not represented in the selected frameworks. Rigor was maintained through inter‐coder agreement within the research team and member checking with study participants. Research reflexivity was implemented using peer debriefing throughout the coding process and reflexive writing to keep an account of the research process and memo personal reflections throughout the study. Collated codes were then categorized into potential themes and subthemes, reviewed, named, and defined to generate a thematic map (Figure 3 and Supporting Information 1–3) (Braun and Clarke 2006). Cross‐group comparative analysis of the themes was implemented, including comparisons within single interviews, within pairs, between different pairs, between the nurse and CBO data, and across different regions (Boeije 2002).

The study results linked participant photos, narratives, and themes. The linked data were then analyzed to identify patterns of perceived climate injustice and climate justice. Findings were compared across the qualitative data and photo locations. The EJ Nursing Framework was utilized to help interpret our data analyses to understand relational pathways that challenge injustices (LeClair, Luebke, and Oakley 2021). In the photos and narratives, nurses are labeled “RN,” and community‐based organizations are labeled “CBO.” Participant numbers were removed to protect confidentiality. In the results, “Interview” refers to data from the semi‐structured interviews, and “Photovoice Narrative” refers to PPM data.

## 3 Findings

Eight nurses and five CBO partners enrolled in this study. The nurses either worked in academic settings or for non‐profits.

### 3.1 Themes

Fifteen broad themes were identified from 21 photovoice and semi‐structured interview transcripts and 49 photographs. All the themes reflect an agreement between CBO representatives and nurses, meaning no themes were identified that reflect only nurse or CBO representative perspectives. As the themes were identified, it became clear that they could be grouped into three categories: Climate Injustice, Climate Justice, and the Transition from Climate Injustice to Climate Justice. The themes were identified inductively from the data and deductively from the guiding frameworks. Study participants described multiple aspects of the themes during their interviews, and the first author named these diverse aspects “subthemes” throughout the data analysis. The eight Climate Injustice themes, sub‐themes, and definitions are listed in Supporting Information 1. The Transition from Injustice to Justice category had one theme in Supporting Information 2. The six Climate Justice themes, definitions, and sub‐themes identified are listed in Supporting Information 3. The Supporting Information also includes the amount of collated data and the number of CBO representatives and nurses who provided the evidence. The fifteen themes are organized and illustrated in the Climate Justice Perspectives and Experiences of Nurses and Their Community Partners Thematic Map (Figure 3). The thematic map displays the characteristics of individuals, communities, and society and the root causes of public health outcomes. The levels are interactive and reinforcing, so they have a cumulative effect on health and well‐being.

Participatory photo mapping results for each of the five partnerships and three solo nurses are displayed in *Climate Injustice* (https://www.google.com/maps/d/u/2/edit?mid=1kHU6a9YMfqt3zvVPkT6MiE5uIDiZl14&usp=sharing), *Transition* (https://www.google.com/maps/d/u/2/edit?mid=1z6gBxLURRvpsuOfZ1aY_iHZwws2xJcg&usp=sharing), and *Climate Justice* (https://www.google.com/maps/d/u/2/edit?mid=1FXxoYyLvtJn2sG8BCoGWv7oJQARrxyk&usp=sharing) maps with the participant photos and associated narratives to illustrate the thematic findings further. Results are presented in the following order: descriptions of the theme and subthemes with example excerpts and a discussion of how the theme was created. Theme names and quotes are presented in italics.

### 3.2 Climate Injustice

#### 3.2.1 Severance From Nature

The first theme, Severance from Nature, speaks of a wound from the colonial severance of relationships within Nature (Supporting Information 1). Nature is capitalized in this article to reflect Indigenous ways of recognizing the personhood of all beings (Kimmerer 2020). The following RN quote depicts this theme: “I feel like there is this inherent wounding from the severance of our relationship with Mother Earth.” Participants described Severance from Nature as a loss of knowledge among people with settler‐colonial ancestry that all life is related and interconnected. Participants shared that, broadly speaking, Indigenous Peoples have retained the knowledge of living in harmony within natural systems. The following quote illustrates this theme of the colonial paradigm being in disharmony with Nature:

…being a Native American person, it's really—I feel like I have a unique perspective and a unique stance in the community of climate justice and planetary health, where it's, you know, we've existed on the Earth for a very long time within these Indigenous systems in harmony, and outside of the Indigenous systems, there is disharmony.

RN Interview

Some participants shared concerns that, due to climate injustices, their Indigenous communities are also experiencing a loss of cultural connections with the land.

Subthemes from the participant data highlight the overarching paradigm of colonial disconnection from Nature as a hierarchical cultural worldview integrated into systems and policies and validating human domination over other beings. Participants noted that this colonial hierarchical paradigm, which promotes a belief in human separation from Nature, allows for desensitization of colonial violence toward all life within Nature. *Severance from Nature* also fosters a numbness among colonizers to the suffering of Indigenous Peoples. While the Social Ecological Model includes a cultural layer (Golden and Wendel 2020), the guiding EJ Nursing Framework does not (LeClair, Luebke, and Oakley 2021). Therefore, the theme was identified through inductively coding the data.

#### 3.2.2 Supremacy

Participants described Supremacy as the patterns of domination that interconnect and reinforce each other (Supporting Information 1). The Supremacy theme deductively emerged from the data through guidance from the EJ Nursing Framework (LeClair, Luebke, and Oakley 2021). Subthemes were identified both inductively from participant perspectives and deductively from the framework (Supporting Information 1).

Most participants named environmental racism a root cause of how frontline and fenceline communities are formed and described how it challenged their ability to address environmental degradation:

It's a systemic issue. Racism is embedded, whether we like it or not, and that has to be illuminated also. Because who gets the brunt of air pollution, water pollution, and land pollution? The BIPOC [Black, Indigenous, and other People of Color] population.

RN Interview

Classism in white fenceline communities was also highlighted because polluting industries targeted low‐income communities (e.g., “white trash”) due to having less economic and political power. Many participants noted that white and wealthy communities were not fenceline to industrial pollution or on the frontlines of climate health effects.

Colonialism and cis‐heteropatriarchy were highlighted as intersecting patterns of domination. For example, “patriarchy” is a concept from the framework, while “cis‐heteropatriarchy” emerged through participant discussions on honoring Two‐Spirit individuals and the role of reproductive justice within environmental justice, particularly in the context of the Eurocentric gender and sexual binaries, cultural loss, genocidal policies, and land dispossession (Ellasante 2021; Indigenous Environmental Network [IEN] 2024). This connection is even more pronounced for Indigenous communities due to their close relationships with the land and the risks of reproductive harm associated with land dispossession, extraction, and other sources of pollution (Jubinville et al. 2024).

Our kind of connection to environmental justice is more cultural than anything…It's directly connected to things like reproductive justice because with our cultural values around, you know, reproductive justice in the Native Community is much more than just access to abortion…It is honoring our Two‐Spirit relatives, and I, as a Two‐Spirit person, I say that for myself and everyone else in our community. It's honoring the ways—I've known others who have been sterilized. So, there's a lot of things that are directly connected to the land and the ways—even the boarding school healing and justice work. The fact that we had federal policy—genocidal policy—that took children away, that is anti‐choice as it gets. And abusing these children, basically torturing them. And so, when it comes to things such as environmental justice, it is directly linked to that. And I think what people may view as environmental justice out there, all that is very important, you know, the birds, the polar bears, the caribou, the clean air for every—for folks that live wherever because we're all affected by it. But we have so many battles to fight…

CBO Interview

Cis‐heteropatriarchal and colonial structures not only marginalize and displace Indigenous communities but also directly contribute to environmental degradation. Thus, climate injustice is inseparable from reproductive justice as each represents a mode through which structures of supremacy perpetuate harm to people and the land. Participants who identified as Indigenous and non‐Indigenous in their interviews described climate injustice as a product of power differentials between colonial systems dominated by men, ongoing trauma and reproductive injustice suffered by women, and their attempts to regain power:

This is not something that just started happening, that this has been happening since the earliest colonial contact that our people have been, you know, victimized, and MMIW [Missing and Murdered Indigenous Women] has been going on for hundreds and hundreds of years. It really goes back to differences in worldviews between colonial settlers and Indigenous peoples. We were traditionally matriarchal, or egalitarian. Our people, our women have always held positions of leadership and power…and I've, and you know, that was seen as extremely backward, bizarre, threatening to colonial people, and so, you know, Indigenous women really became highly sexualized and were expected to, you know, be submissive or be used as sex‐slaves, just really atrocious things. You know, over the hundreds of years since colonization, there's been federal legislation and land disposition and forced removal, bordering schools, basically assimilation and termination policies that were enacted to continue to try to assimilate Indigenous peoples into Eurocentric ways.

RN Interview

So you know, again, it is this patriarchal—these attitudes—and even women in positions of power here—there's not. It's annoying, but it's satisfying knowing that when we roll up [laughs] that they're not happy because every time we've rolled up to a city council meeting, we've had, you know, we've had something that is an issue. And they can scream, “You know they're liars…” blah blah blah, but we've got black and white documentation, empirical scientific data, etc., to back up our claims, and they've got nothing. So, you know, while it's insulting, it's also satisfying knowing that as soon as we hit the door, and they see us, that we're basically watching them being emasculated [laughs]. They hate us. Like it's hilarious.

CBO Interview

#### 3.2.3 Corporate Capitalism

In *Corporate Capitalism*, corporations are not expected to work for the social good but instead create profit for their own self‐interest (Supporting Information 1) (Kasser et al. 2007). The following quote illustrates this theme:

They call it ‘The Resource Curse.’ Like where we live, the [Name] Valley has so many natural resources…and it's fueled generations, but it's come at a major cost.

RN Photovoice Narrative for Acid Mine Drainage photo

Participant perspectives aligned with racial capitalism as a subtheme (Supporting Information 1). The literature defines racial capitalism as the extraction and exploitation of land and racialized people, leading to the depletion of all life, which widens inequities for Black, Indigenous, and other racialized people who are forced to the frontlines and fencelines of corporate industrialized projects (Laster Pirtle 2020). Many participants shared how corporations exploit and extract from marginalized populations. In addition to racialized communities, participants discussed the “willful ignorance” of low‐income white, “blue‐collar” communities, such as those in Appalachia, exploited by corporations that offer employment but omit information on toxic exposures that damage worker and community health. Participants noted community socioeconomic status as a significant barrier to fighting industry lobbyists hired to protect corporate welfare over public health.

This theme was deductively identified through guidance from the “Capitalism” concept in the EJ Nursing Framework (LeClair, Luebke, and Oakley 2021). The lead author is familiar with the concept of “Corporate Capitalism” from the literature, and participant perspectives aligned with that specific aspect of capitalism; therefore, the concept of “Capitalism” from the EJ Framework was narrowed to “Corporate Capitalism” in this study. Subthemes were inductively identified (Supporting Information 1).

#### 3.2.4 Corporate Climate Pollution

Participants noted many aspects of Corporate Climate Pollution, such as “legacy pollution,” which denotes the longstanding effects of toxic waste exposure from corporate industries (Supporting Information 1). Participants shared stories about industry workers who voiced concerns about their health being threatened and fear of losing their employment. Multiple study participants who work to advance climate justice in their communities, including nurses and CBO representatives, were threatened by corporations and governments protecting them. Participants used the term “sacrifice zone” to describe how their community's health is sacrificed to support the welfare of the corporate industry:

It's a term that is used to describe a place like ours because we don't really see the real benefits to these companies. It's really the people at the very, very top. The people in Thailand, you know, running the company… [Corporation Name], same thing. They don't really live around here, the owners and stuff… the shareholders and CEOs and stuff, so we are the sacrifice for their profit.

RN Photovoice Narrative for Cracker Banner photo

This theme and subthemes were inductively identified from the study participants' stories (Supporting Information 1).

#### 3.2.5 State Violence

Participants described many aspects of State Violence, noting that it is enacted through government partnerships with corporations to allow pollution, neglecting community protections, and refusing to hold corporations accountable for ecocide (Supporting Information 1). Ecocide is unlawful or reckless acts committed with knowledge of severe and long‐term damage to the environment being caused by those acts (Stop Ecocide Foundation 2021). Participants did not believe their governments prioritized climate justice funding and shared how inadequate laws, policies, and regulations rendered sacrifice zones invisible.

Participants discussed how government agencies and politicians protect themselves through the lack of transparent communication about health risks and/or partnering with the media to perpetuate harmful stories that provide a false sense of safety and security. Some participants in the study felt that the federal government is “co‐opting” the climate justice movement to promote a false sense of inclusion and accountability. Nurses and their community partners shared experiences of government officials and politicians engaging in surveillance and threatening whistle‐blowers, including their staff members and community organizers.

The quote below depicts the effects of government neglect, an aspect of *State Violence* shared by multiple participants in frontline and fenceline communities:

We rely on our agencies to be our advocates, and in this instance, every single agency that was supposed to advocate for the community failed. Every single one of them, either because they wanted to, they didn't care, or dereliction of duty, but every single agency failed to advocate for the community and protect the citizens of the community.

CBO Narrative for Sock Grinder photo

The theme of *State Violence* was deductively identified through guidance from the “Violence” concept in the EJ Nursing Framework (LeClair, Luebke, and Oakley 2021). The lead author is familiar with the concept of “State Violence” from the literature, (Kojola and Pellow 2021; Mushonga 2022) and participant perspectives aligned with the concept; therefore, the concept of “Violence” was narrowed to “State Violence” in this study.

#### 3.2.6 Mortality

Participant interviews also highlight that Mortality is a multispecies and intergenerational concern in their communities (Supporting Information 1). Participant stories, especially from those in fenceline communities, discussed death from cancers and abandoned mine shafts that residents fall into. Participants described the interconnected aspects of Mortality among humans and other species in their community.

It's 2023, there's still injustice going on. This site is still not clean, you know, the tribe is still dying from the effects of this dumping, of these toxins, you know, the animals have cancer, the fish are toxic, you know, the water, the air, the soil…

RN Interview

The theme of *Mortality* was deductively identified through guidance from the “Mortality” concept in the EJ Nursing Framework (LeClair, Luebke, and Oakley 2021).

#### 3.2.7 Morbidity

Study participants highlighted respiratory, bone, neurological, and heat‐related diseases, among many others, as examples of Morbidity in their communities (Supporting Information 1). Participants shared that Morbidity, like Mortality, encompasses multispecies and intergenerational experiences. Participants expressed that frontline and fenceline community members have a dangerous lack of understanding about the relationship between their health and environmental degradation:

…getting hit with heat and smoke at that time as well, so it was kind of like a triple threat respiratory crisis in those communities, and it was hard to tell if people were getting ill from the wildfire smoke exposure or from COVID. And some people were told, you know, that if they were having—they got the vaccine, and then there would be a smoky day, and then they would feel sick, and then they would say, ‘Oh, it's the vaccine.’ No, it's the smoke.

RN Interview

The theme of *Morbidity* was deductively identified through guidance from the “Morbidity” concept in the EJ Nursing Framework (LeClair, Luebke, and Oakley 2021).

#### 3.2.8 Despair

Study participants highlighted multiple aspects of Despair, including fear of the unknown among community members, such as the loss of agricultural work due to climate impacts (Supporting Information 1). A sense of powerlessness was mentioned, including a deep concern for future generations. Nurses and CBO representatives also noted a strong dissociation among community members who experience ongoing toxic exposures—especially those that span decades or longer. For example, participants described the sense of Despair about the loss of spiritual connections within Nature for many people who identify as Indigenous. Nurse study participants who were not members of the fenceline or frontline communities they partnered with experienced secondary traumatic or moral distress. As one RN explained,

That's the other unwritten part of this work, right? That the struggle is long, dangerous, and probably going to be disappointing, not to get too gloomy… we fail a lot, right? We get tired because the challenges are insurmountable.

The theme of *Despair* was deductively identified through guidance from the “Despair” concept in the EJ Nursing Framework (LeClair, Luebke, and Oakley 2021).

### 3.3 The Transition From Injustice to Justice

#### 3.3.1 Long Struggle

The theme Long Struggle signifies the struggle of frontline and fenceline communities over a long period of time in their fight for climate justice (Supporting Information 2). Participants illuminated aspects of the Long Struggle, such as how navigating the previously described toxic and abusive legacies can be arduous, isolating work where nurses and their community partners feel like tiny forces that must dismantle enormous structures. Participants shared that the struggle is hard and messy on the journey toward climate justice. The dangers of State Violence and Corporate Climate Pollution appear interconnected with the Long Struggle, as the nurse and CBO representative study participants who identified as members of the frontline and fenceline communities they served described how they must constantly navigate their safety, health, and well‐being. Participants also highlighted the importance of retaining a cultural memory of the traumatic stories of injustices so that they can serve as lessons for future generations. The following quotes demonstrate this theme:

You've been fighting that good fight for a long time, and you're a weary soldier.

CBO Interview

You are told that the struggle is not going to be worth it. You are told that you are going to lose… one of the chilling interviews was a conversation with an environmental lawyer, I probably talked about it before, you know, she basically said, you know, ‘they will win most of the time. Ninety‐five percent of the time. Five percent of the time, you will win. So, ninety‐five percent of the time, you will lose.’ How do you actually stay in this? Because it's not the glory road…So, this is like, we cannot win, right? So, if you do that, then my good friend and mentor [NAME] says, ‘If you're not in the struggle, you're not living.’ The struggle is where you have to stay… It's really about staying in the struggle and staying with people in solidarity in the struggle.

RN Interview

This theme and its subthemes were guided inductively by the stories of participants in this study (Supporting Information 2).

### 3.4 Climate Justice

All the themes and subthemes in the Climate Justice category were inductively guided by the stories participants shared.

#### 3.4.1 Spiritual Relationships Within Nature

The climate justice theme, Spiritual Relationships Within Nature, refers to participants’ experiences of finding spiritual meanings and healing relationships within Nature (Supporting Information 3). These experiences are retained, remembered, or regained through the Long Struggle toward climate justice. Aspects of this theme identified from participant interviews included healing settler colonialism and finding spiritual connections within Nature. Shifting the cultural paradigm to one that prioritizes Indigenous worldviews for current and future generations is another subtheme.

Healing relationships with more‐than‐human relatives was a common subtheme for nurse and CBO representative participants. Relationships were common with land and water systems and other species. One CBO noted that Nature can have a “powerful impact on your spirit.” Participants gained a sense of resiliency from *Spiritual Relationships Within Nature*. Many shared that through adversity and struggles, Indigenous Peoples and their interconnected ecosystems are still here and thriving. The following quote demonstrates this subtheme:

We felt like we were able to not only grow nutritious vegetables, and to give the—you know, there's a whole bunch of other things that go on. There's that spiritual side, that reconnection with the land. It's having some people from the community come out to the farm and becoming engaged in that, and getting away from their funky little toxic neighborhood that they live in every day so that spiritual side starts to develop again or reconnect again. And then, you know, we're able to provide that healthy food for them too, as well, you know, which again, is making us feel better about being able to do something, even though it's on a small scale. It's doing something, and something is better than nothing…I think all Indigenous nations are—really have been, you know, trying to deal with the assimilation and the colonialism that has happened, and it's been very devastating, but I think we're all trying to reconnect with our true selves again, and I think that you know, part of it is that we're always going to be searching for our homelands, our culture, our language and to get back to being who we are supposed to be and I think it's going to be a tremendous—it's gonna take a tremendous amount of time. But I don't think a lot of us see it in that way. I think we see it as a sense of a homecoming back to our DNA, our true self.

CBO Photovoice narrative with Book photo.

#### 3.4.2 Belonging

Aspects of Belonging from participant stories include increased social capital, multicultural and intergenerational connection, and having a sense of place (Supporting Information 3). Study participants reflected on the importance of having places to gather and build community relationships and power:

You had a very nice mix of tribal members and non‐tribal members, and people who go to the church and people who don't go to church. So, it was just a beautiful socialization, and a coming‐together, if you will…

RN Interview

Places within Nature, such as community gardens, were identified as common sites to gather residents who might not normally interact, such as children and elders. There was also a recognition of *Belonging* and *Spiritual Relationships Within Nature* as mutually reinforcing because having a sense of *Belonging* with more‐than‐human relatives and having places to “just breathe” in Nature were critical for health and well‐being. Participants shared that fostering a sense of *Belonging* for community members was essential for building a climate justice movement.

#### 3.4.3 Abundance

Participants shared stories about Abundance that described how local, regenerative economies were supported through community member engagement with natural and built environments (Supporting Information 3). Communities that created farms and gardens had sustainable sources of nourishment and increased access to abundant greenspace and wildlife for community member engagement and education, supporting Belonging:

We hired youth to maintain the land and hired youth as landscape educators about the open space…it gave people economic opportunities to earn money as well as socialization for people to come together.

CBO

Study participants also viewed the challenge of climate mitigation and resilience as opportunities to build and support their local economy.

#### 3.4.4 Protected Communities

Public health was protected and assured in Protected Communities (Supporting Information 3). Aspects derived from participant stories include political power and solidarity within and between frontline and fenceline communities and their allies. Participants shared that community members can easily access public health information in Protected Communities. Essential subthemes of Protected Communities also included government agencies assuring corporate responsibility for pollution prevention and accountability for cleaning historical pollution. Participants further expanded the notion of “community” to include more‐than‐humans, reflecting that all species within ecosystems experience climate injustices and that the health of all life must be valued. Promoting the rights of Nature is further highlighted in this theme of Protected Communities:

[CBO] saw these children disturbing sediments that people said, “Well, there's dioxins down there. There's poison down there,” and all of a sudden, it becomes this idea that it's not only protecting the salmon, but what we're doing here is environmental systems protecting people, and how do we begin to have a turn, and begin to think about the protection of people and that, I think, that's a real climate justice moment, right?

RN Photovoice Narrative for The “Birthing” Channel photo

#### 3.4.5 Communities of Care

Communities of Care subthemes include how it is multi‐cultural,—generational, and—species (Supporting Information 3). Study participants were inspired by the care youth in their communities showed toward fellow community members and their environments. They also found the ability to persevere when they worked in solidarity with others, including allies not from the community. Participants recognized that everyone has a unique gift to apply within Communities of Care. Participants also shared visions of advancing the movement through support networks between frontline and fenceline communities across the United States to help accelerate climate resilience. Caring for communities and caring for the Earth were described as interconnected:

It reminds me of, like these concentric circles, of what environmental justice can look like. It starts here—maybe that's me right here in this tiny little dot. And then this little circle is my friends, that I combine my efforts with to do this project and this vegan food prep for the houseless, and it reverberates to the community within the houseless people, and then it reverberates out where people from total strangers were trying to get involved and to donate food and to donate clothes and clean water and gasoline for generators, and it just ended up becoming this bigger and bigger and bigger thing.

RN Photovoice Narrative for Beet photo.

#### 3.4.6 Planetary Health and Well‐Being

Aspects of Planetary Health and Well‐being derived from participant stories include the interconnection of body‐mind‐spirit within Nature, climate resilience, healing from historical trauma, and hope for future generations (Supporting Information 3). Participants determined the Planetary Health and Well‐being theme to be both an outcome and driving force of the climate justice movement:

The driving force, I guess, of the community is, like, how can we help planetary health? And it's a very close second is the social health of humanity. And how does that, like, how do they play together? And how can we do better in the world? If you have your number one driving force is the health of the planet because, without the health of the planet, health of humans doesn't matter as much.

RN Photovoice Narrative for Beet photo.

## 4 Discussion

This is the first study to explore how nurses and their CBO partners envision, perceive, and experience climate justice in frontline and fenceline communities using a novel mixed‐methods approach of participatory photo mapping. Through photovoice and semi‐structured interviews, participants described a broad understanding of climate justice to encompass perspectives on planetary health inequities, such as overexposure to pollution and loss of relationships within Nature. These perspectives align with calls from the research community and the United Nations’ declaration of humanity's three greatest interlinked global public health threats of climate change, pollution, and threats to Nature—known as the “triple planetary crisis” (Abbasi et al. 2024; Passarelli, Denton, and Day 2021; United Nations Environment Programme 2021). This crisis was perceived in this study as resulting from colonial severance from Nature, the supremacy of some people over others, and corporate pollution perpetrated in collusion with governments. Study participants described how these complex, oppressive forces caused increased mortality, morbidity, and despair in their communities. Moving toward planetary health was experienced as regaining spiritual relationships within Nature and fostering a sense of belonging, protection, and care in communities.

From the analysis, the EJ Nursing Framework was relevant to guiding the deductive coding as it aligned with participant experiences and is thus a valuable approach for future researchers (LeClair, Luebke, and Oakley 2021). However, results implied support for expanding the EJ Nursing Framework to include a cultural worldview layer (i.e., *Severance from Nature*) to inform and holistically guide research in climate justice. This finding aligns with other frameworks being applied in climate justice and planetary health research, such as the Planetary Health Education Framework, which includes the Interconnection Within Nature as a central domain, and the Just Transition Framework, which promotes a culture based on caring and sacredness of relationships with people and the planet (Guzmán et al. 2021; Just Transition Alliance 1997). Furthermore, all the themes in the Climate Justice category were identified inductively from the data, as the EJ Nursing Framework focused primarily on injustices. An expanded version of the EJ Nursing Framework could address themes in the Climate Justice category of the thematic map.

Nurses and other public health professionals are expected to partner with communities to advance climate justice and planetary health, and this study provides early exploratory evidence of current perceptions and lived experiences (ANA 2022; APHA 2013; Council on Linkages Between Academia and Public Health Practice 2021; Evans‐Agnew, LeClair, and Sheppard 2024; Kalogirou, Olson, and Davidson 2020; Kulbok et al. 2012). Future research could explore more broadly how CBO representatives perceive the root causes of planetary health injustices and related public health outcomes and their perspectives on the utility of nurse‐community partnerships to advance planetary health. Research is also needed to understand the barriers and facilitators to addressing climate injustice and promoting climate justice in government public health and other settings where nurses practice.

The devastation that the colonial cultural worldview of *Severance from Nature* brought to communities in this study was profound in how it allowed some humans to dominate other humans and species through acts of *Supremacy*. It also allowed *Corporate Capitalism* to flourish by valuing profits over public health. The health outcomes described by participants in this study align with emerging nursing research on the pathways and processes of embodying trauma secondary to settler colonialism (Thomas et al. 2023). This study revealed how nurses and their CBO partners perceived the need to transform the worldview of *Severance from Nature* into a worldview that promotes *Spiritual Relationships Within Nature*. Regaining these relationships and interconnection with all life helped participants feel a sense of *Belonging* with other community members and the places where they lived. For this to happen, findings from this study suggest the importance of expanding nurses’ understanding of community to include relationships with other species outside of humans. Participants stressed that this shift in perspective must center on *Planetary Health and Well‐being* as the driving forces for climate justice. These perspectives are shared by Indigenous scientists, educators, and leaders worldwide (Redvers et al. 2022). In this study, CBO representatives and nurses who experienced climate justice described their communities as abundant and protected places where residents cared for each other and all life.

While spirituality is not new to the nursing discipline (Willis and Leone‐Sheehan 2019), further research could explore what incorporating a worldview that promotes spiritual relationships within Nature means for Indigenous and non‐Indigenous nurses and their CBO partners. For example, how can non‐Indigenous nurses promote a cultural shift in the worldview of the future nursing workforce without appropriating Indigenous spirituality? The need for this shift in worldview from anthropocentric (i.e., human‐centered) to kincentric (i.e., relationships with more‐than‐human kin) and posthumanist (i.e., questioning the boundaries between humans, more‐than‐humans, and Nature) is increasingly reflected in the nursing literature (Dillard‐Wright, Walsh, and Brown 2020, 2024; Evans‐Agnew, LeClair, and Sheppard 2024; LeClair 2021; Perry 2024). The term more‐than‐human refers to all species and life in addition to humans and prioritizes the relational value beyond being of instrumental use to humans (Abram 1996). The Mi'kmaw concept of *Etuaptmumk* (Two‐Eyed Seeing) and the Māori Nursing theory of Cultural Safety could assist this exploration (Bartlett, Marshall, and Marshall 2012; Ramsden 2002). *Etuaptmumk* weaves Indigenous and Western worldviews and knowledge systems to co‐learn and co‐create solutions for environmental crises (Bartlett, Marshall, and Marshall 2012). Cultural Safety prompts nurses to examine their cultural realities, consider the socio‐political contexts, and build relations that those they care for deem “safe” (Ramsden 2002). Members of the Alliance of Nurses for Healthy Environments utilized these concepts in developing the Global Nurse Agenda for Climate Justice (Climate Justice in Nursing Steering Committee 2021) and shared exemplars for incorporating *Etuaptmumk* and Cultural Safety into current climate justice practice and guidance in future nursing research (Evans‐Agnew, LeClair, and Sheppard 2024).

The findings from this study could be used as evidence to help inform nursing's response to calls for advocacy, education, and practice to support climate justice and planetary health (ANA 2022; Evans‐Agnew, LeClair, and Sheppard 2024; Kalogirou, Olson, and Davidson 2020; Kurth 2017; LeClair, Evans‐Agnew, and Cook 2022; LeClair and Potter 2022; Lilienfeld et al. 2018; Nicholas and Breakey 2017; Travers et al. 2019). The thematic map highlights interactive and reinforcing layers of climate injustice and justice, depicting cumulative effects on health (Figure 3). Therefore, nurses who want to partner with communities to advocate for climate justice must concurrently address factors identified at multiple levels. For example, communities across the U.S. and Canada are heavily affected by dangerous air quality from the intersection of pollution and wildfires. Nurses working in these communities addressing increasing respiratory problems (i.e., *Morbidity*) could also support fenceline communities in their advocacy to hold government and corporate industries accountable for climate pollution. The results from this study indicate that fenceline communities have critical perspectives and experiences in the long struggle for clean air, and participants expressed interest in building solidarity between frontline and fenceline communities through mutual advocacy for climate justice. While the concept of state violence is recognized in nursing literature for its relationship with the colonial, racist, misogynist, trans‐ and homophobic origins of policing (Paynter et al. 2021), findings from this study suggest that nurses working to advance climate justice and planetary health can expand this understanding of state violence to include the collusion of governments and corporations in the sacrifice of public health for corporate welfare. Educators who want to include climate justice and planetary health in public health and nursing curricula could consider integrating discussions on these topics to promote a shift in the perspectives and worldviews of future nursing workforce members.

## 5 Study Limitations

This study had several limitations. First, while the sample size was within the recommended range for this type of mixed methods study, it was limited; thus, there may have been other perspectives and experiences not represented in these findings. We did not ask participants to disclose their social identities, although many openly shared this information during interviews. Future studies should attempt to increase the sample through alternative recruitment methods and document participants’ social identities to better inform the data analysis. Additional or different themes might also emerge from participants in more diverse geographic settings. This study represented the work of nurses in academic and non‐profit settings in the United States, and the results cannot necessarily be applied to nurses working in other settings or countries. This study misses the perspectives and experiences of nurses working in government and other settings responsible for assessing and assuring population health equity. Finally, while we remained open to inductively identifying themes from the data, using preexisting frameworks, such as the EJ Nursing Framework, to guide analysis can have limitations in providing a “lens” through which to read and code the data and develop themes, which may not fully reflect all the data (Braun and Clarke 2022).

## 6 Conclusion

This study described how nurses and their community partners envision, perceive, and experience climate justice in frontline and fenceline communities. Climate injustice was perceived as resulting from colonial severance from Nature, the supremacy of some people over others, corporate capitalism, and ongoing climate pollution perpetuated by corporations and governments. Study participants described how these phenomena caused increased incidences of mortality, morbidity, and despair in their communities. Nurses and their community partners experienced transitioning from climate injustice to climate justice as a long, hard struggle. Climate justice was envisioned as regaining, remembering, or retaining spiritual relationships within Nature and fostering a sense of belonging, protection, and care in communities. Participants perceived planetary health and well‐being as the outcomes and driving forces of climate justice in frontline and fenceline communities. Understanding these visions, perspectives, and experiences can inform strategies to support climate justice through authentic nurse‐community partnerships. This article aims to spark continued dialogue and action among nurses and their community partners who experience climate injustice and persist through solidarity in the long struggle for justice.

## Ethics

The University of Wisconsin‐Madison Minimal Risk Research IRB determined the study met the exempt criteria. Information about the reasons for doing the research was shared with all participants in the Research Information and Consent document. All participants could ask questions during the consent and enrollment process and at any time during their participation in the study.

## Conflicts of Interest

The authors declare no conflicts of interest.

## Supporting information

Supporting information.

## Data Availability

The data are unavailable due to privacy or ethical restrictions.

## References

[nin12690-bib-0001] Abbasi, K. , P. Ali , V. Barbour , et al. 2024. “Time to Treat the Climate and Nature Crisis As One Indivisible Global Health Emergency.” Nursing Inquiry 31, no. 1, Article: e12612. 10.1111/nin.12612. PMC11998606 40237015

[nin12690-bib-0002] Abram, D. 1996. The Spell of the Sensuous: Perception and Language in a More‐Than‐Human World. New York, NY: Pantheon Books.

[nin12690-bib-0003] ANA . 2022. Public Health Nursing: Scope and Standards of Practice, 3rd ed. Silver Spring, MD: American Nurses Association.

[nin12690-bib-0004] ANA . 2023. Nurses' Role in Addressing Global Climate Change, Climate Justice, and Planetary Health. Silver Spring, MD: American Nurses Association. https://www.nursingworld.org/practice-policy/nursing-excellence/official-position-statements/id/climate-change/.

[nin12690-bib-0005] APHA . 2013. The Definition and Practice of Public Health Nursing: A Statement of the Public Health Nursing Section. Washington, DC: American Public Health Association. https://www.apha.org/~/media/files/pdf/membergroups/phn/nursingdefinition.ashx.

[nin12690-bib-0006] Baptista, A. I. , S. Jesudason , M. Greenberg , and A. Perovich . 2023. “Landscape Assessment of the Us Environmental Justice Movement: Transformative Strategies for Climate Justice.” Environmental Justice 16, no. 2: 111–117. 10.1089/env.2021.0075.

[nin12690-bib-0007] Bartlett, C. , M. Marshall , and A. Marshall . 2012. “Two‐Eyed Seeing and Other Lessons Learned Within a Co‐Learning Journey of Bringing Together Indigenous and Mainstream Knowledges and Ways of Knowing.” Journal of Environmental Studies and Sciences 2, no. 4: 331–340. 10.1007/s13412-012-0086-8.

[nin12690-bib-0008] Battle, C. P. 2020. “Chapter 8: Rise; An Offering From the Bayou.” In All We Can Save, Edited by A. E. Johnson , and K. K. Wilkinson , 329–333. New York, NY: One World.

[nin12690-bib-0009] Bekkar, B. , S. Pacheco , R. Basu , and N. DeNicola . 2020. “Association of Air Pollution and Heat Exposure With Preterm Birth, Low Birth Weight, and Stillbirth in the US: A Systematic Review.” JAMA Network Open 3, no. 6: e208243. 10.1001/jamanetworkopen.2020.8243. 32556259 PMC7303808

[nin12690-bib-0010] Benz, S. A. , and J. A. Burney . 2021. “Widespread Race and Class Disparities in Surface Urban Heat Extremes Across the United States.” Earth's Future 9, no. 7: e2021EF002016. 10.1029/2021ef002016.

[nin12690-bib-0011] Boeije, H. 2002. “A Purposeful Approach to the Constant Comparative Method in the Analysis of Qualitative Interviews.” Quality & Quantity 36: 391–409. 10.1023/A:1020909529486.

[nin12690-bib-0012] Braun, V. , and V. Clarke . 2006. “Using Thematic Analysis in Psychology.” Qualitative Research in Psychology 3: 77–101. 10.1191/1478088706qp063oa.

[nin12690-bib-0013] Braun, V. , and V. Clarke . 2019. “Reflecting on Reflexive Thematic Analysis.” Qualitative Research in Sport, Exercise and Health 11, no. 4: 589–597. 10.1080/2159676X.2019.1628806.

[nin12690-bib-0014] Braun, V. , and V. Clarke . 2022. Thematic Analysis: A Practical Guide. London: Sage.

[nin12690-bib-0015] Bullard, R. 1990. Dumping in Dixie: Race, Class, and Environmental Quality. Westview Press.

[nin12690-bib-0016] Bullard, R. 1994. “The Legacy of American Apartheid and Environmental Racism.” Journal of Civil Rights and Economic Development 9, no. 2: 445–474.

[nin12690-bib-0017] Climate Justice in Nursing Steering Committee . 2021. *Climate Justice Agenda for Nursing*. Alliance of Nurses for Healthy Environments. https://envirn.org/climate-change/climate-justice-agenda-for-nursing/.

[nin12690-bib-0018] Council on Linkages Between Academia and Public Health Practice . 2021. Core Competencies for Public Health Professionals. Council on Linkages Between Academia and Public Health Practice. https://www.phf.org/resourcestools/Documents/Core_Competencies_for_Public_Health_Professionals_2021October.pdf.

[nin12690-bib-0019] Curley, A. , and M. Lister . 2020. “Already Existing Dystopias: Tribal Sovereignty, Extraction, and Decolonizing the Anthropocene.” In Handbook on the Changing Geographies of the State: New Spaces of Geopolitics, edited by S. Moisio , N. Koch , A. E. Jonas , and C. Lizotte , 251–262. Cheltenham, UK: Edward Elgar Publishing.

[nin12690-bib-0020] Dennis SF, Jr , S. Gaulocher , R. M. Carpiano , and D. Brown . 2009. “Participatory Photo Mapping (PPM): Exploring an Integrated Method for Health and Place Research With Young People.” Health & Place 15, no. 2: 466–473. 10.1016/j.healthplace.2008.08.004. 18930431

[nin12690-bib-0021] Dillard‐Wright, J. , J. H. Walsh , and B. B. Brown . 2020. “We Have Never Been Nurses: Nursing in the Anthropocene, Undoing the Capitalocene.” Advances in Nursing Science 43, no. 2: 132–146. 10.1097/ANS.0000000000000313. 32345801

[nin12690-bib-0022] Dillard‐Wright, J. , J. B. Smith , J. Hopkins‐Walsh , E. Willis , B. B. Brown , and E. C. Tedjasukmana . 2024. “Notes on [Post]Human Nursing: What It Might Be, What It is Not.” Nursing Inquiry 31, no. 1: e12562. 10.1111/nin.12562. 37211658

[nin12690-bib-0023] Ebi, K. L. , J. Vanos , J. W. Baldwin , et al. 2021. “Extreme Weather and Climate Change: Population Health and Health System Implications.” Annual Review of Public Health 42, no. 1: 293–315. 10.1146/annurev-publhealth-012420-105026. PMC9013542 33406378

[nin12690-bib-0024] Ellasante, I. K. 2021. “Radical Sovereignty, Rhetorical Borders, and the Everyday Decolonial Praxis of Indigenous Peoplehood and Two‐Spirit Reclamation.” Ethnic and Racial Studies 44, no. 9: 1507–1526. 10.1080/01419870.2021.1906437.

[nin12690-bib-0025] Evans‐Agnew, R. , J. LeClair , and D.‐A. Sheppard . 2024. “Just‐Relations and Responsibility for Planetary Health: The Global Nurse Agenda for Climate Justice.” Nursing Inquiry 31, no. 1: e12563. 10.1111/nin.12563. 37256546

[nin12690-bib-0026] Garibay, V. , and E. Arevalo . 2017. Advancing Climate Justice in California: Guiding Principles and Recommendations for Policy and Funding Decisions. California, USA: Climate Justice Working Group. https://www.healthyworldforall.org/en/pdf/AdvancingClimateJusticeInCaliforniaWithoutAppendix.pdf.

[nin12690-bib-0027] Golden, T. L. , and M. L. Wendel . 2020. “Public Health's Next Step in Advancing Equity: Re‐Evaluating Epistemological Assumptions to Move Social Determinants From Theory to Practice.” Frontiers in Public Health 8: 131. 10.3389/fpubh.2020.00131. 32457863 PMC7221057

[nin12690-bib-0028] Grineski, S. E. , and T. W. Collins . 2018. “Geographic and Social Disparities in Exposure to Air Neurotoxicants at U.S. Public Schools.” Environmental Research 161: 580–587. 10.1016/j.envres.2017.11.047. 29245126 PMC5760180

[nin12690-bib-0029] Guzmán, C. A. F. , A. A. Aguirre , B. Astle , et al. 2021. “A Framework to Guide Planetary Health Education.” Lancet Planetary Health 5, no. 5: e253–e255. 10.1016/S2542-5196(21)00110-8. 33894134

[nin12690-bib-0030] Hayden, M. H. , P. J. Schramm , C. B. Beard , et al. 2023. “Human Health.” In Fifth National Climate Assessment, edited by A. R. Crimmins , C. W. Avery , D. R. Easterling , K. E. Kunkel , B. C. Stewart , T. K. Maycock , 1–48. Washington, DC: U.S. Global Change Research Program. 10.7930/NCA5.2023.CH15.

[nin12690-bib-0031] Indigenous Environmental Network [IEN] . 2024. “Nex Benedict: A Continued Legacy of Systemic Violence Against Two Spirit Relatives.” https://www.ienearth.org/nex-benedict-a-continued-legacy-of-systemic-violence-against-two-spirit-relatives/.

[nin12690-bib-0032] International Climate Justice Network . 2002. Bali Principles of Climate Justice. Berkeley, CA, USA: Corpwatch: Holding Corporations Accountable. https://www.corpwatch.org/article/bali-principles-climate-justice.

[nin12690-bib-0033] Jubinville, D. , J. Smylie , S. Wolfe , et al. 2024. “Relationships to Land as a Determinant of Wellness for Indigenous Women, Two‐Spirit, Trans, and Gender Diverse People of Reproductive Age in Toronto, Canada.” Canadian Journal of Public Health 115, no. 2: 253–262. 10.17269/s41997-022-00678-w. 36042155 PMC11582287

[nin12690-bib-0034] Just Transition Alliance . 1997. The Just Transition Alliance Definition of a Just Transition and Just Transition Principles. Just Transition Alliance. https://climatejusticealliance.org/wp-content/uploads/2018/06/Just-Transition-Alliance-Just-Transition-Principles.pdf.

[nin12690-bib-0035] Kallio, H. , A. M. Pietilä , M. Johnson , and M. Kangasniemi . 2016. “Systematic Methodological Review: Developing a Framework for a Qualitative Semi‐Structured Interview Guide.” Journal of Advanced Nursing 72, no. 12: 2954–2965. 10.1111/jan.13031. 27221824

[nin12690-bib-0036] Kalogirou, M. R. , J. Olson , and S. Davidson . 2020. “Nursing's Metaparadigm, Climate Change and Planetary Health.” Nursing Inquiry 27, no. 3: e12356. 10.1111/nin.12356. 32519446

[nin12690-bib-0037] Kasser, T. , S. Cohn , A. D. Kanner , and R. M. Ryan . 2007. “Some Costs of American Corporate Capitalism: A Psychological Exploration of Value and Goal Conflicts.” Psychological inquiry 18, no. 1: 1–22. 10.1080/10478400701386579.

[nin12690-bib-0038] Kimmerer, R. 2020. Braiding Sweetgrass: Indigenous Wisdom, Scientific Knowledge and the Teachings of Plants. Minneapolis, MN: Milkweed.

[nin12690-bib-0039] Kojola, E. , and D. N. Pellow . 2021. “New Directions in Environmental Justice Studies: Examining the State and Violence.” Environmental Politics 30, no. 1–2: 100–118. 10.1080/09644016.2020.1836898.

[nin12690-bib-0040] Kulbok, P. , E. Thatcher , E. Park , and P. Meszaros . 2012. “Evolving Public Health Nursing Roles: Focus on Community Participatory Health Promotion and Prevention.” OJIN: The Online Journal of Issues in Nursing 17, no. 2: e10913734. 22686109

[nin12690-bib-0041] Kurth, A. E. 2017. “Planetary Health and the Role of Nursing: A Call to Action.” Journal of Nursing Scholarship 49, no. 6: 598–605. 10.1111/jnu.12343. 28960761

[nin12690-bib-0042] Landrigan, P. J. , M. Britt , S. Fisher , et al. 2024. “Assessing the Human Health Benefits of Climate Mitigation, Pollution Prevention, and Biodiversity Preservation.” Annals of Global Health 90, no. 1: Art: 1. 10.5334/aogh.4161. 38186855 PMC10768568

[nin12690-bib-0043] Laster Pirtle, W. N. 2020. “Racial Capitalism: A Fundamental Cause of Novel Coronavirus (COVID‐19) Pandemic Inequities in the United States.” Health Education & Behavior 47, no. 4: 504–508. 10.1177/1090198120922942. 32338071 PMC7301291

[nin12690-bib-0044] LeClair, J. 2021. “Building Kincentric Awareness in Planetary Health Education: A Rapid Evidence Review.” Creative Nursing 27, no. 4: 231–236. 10.1891/cn-2021-0009. 34903624

[nin12690-bib-0045] LeClair, J. 2023. Climate Justice Perspectives and Strategies Implemented by Public Health Nurses and Their Community Partners. Madison, WI: Doctoral Dissertation, University of Wisconsin‐Madison.

[nin12690-bib-0046] LeClair, J. , R. Evans‐Agnew , and C. Cook . 2022. “Defining Climate Justice in Nursing for Public and Planetary Health.” American Journal of Public Health 112, no. 55: S256–S258. 10.2105/AJPH.2022.306867. 35679549 PMC9184902

[nin12690-bib-0047] LeClair, J. , J. Luebke , and L. D. Oakley . 2021. “Critical Environmental Justice Nursing for Planetary Health: A Guiding Framework.” *Advances in Nursing Science* . Advance online publication. 10.1097/ANS.0000000000000398. 34569987

[nin12690-bib-0048] LeClair, J. , and T. Potter . 2022. “Planetary Health Nursing.” AJN, American Journal of Nursing 122, no. 4: 47–52. 10.1097/01.NAJ.0000827336.29891.9b. 35348518

[nin12690-bib-0049] LeClair, J. , T. Watts , and S. Zahner . 2021. “Nursing Strategies for Environmental Justice: A Scoping Review.” Public Health Nursing 38, no. 2: 296–308. 10.1111/phn.12840. 33210747

[nin12690-bib-0050] Lilienfeld, E. , P. K. Nicholas , S. Breakey , and I. B. Corless . 2018. “Addressing Climate Change Through a Nursing Lens Within the Framework of the United Nations Sustainable Development Goals.” Nursing Outlook 66, no. 5: 482–494. 10.1016/j.outlook.2018.06.010. 30172574

[nin12690-bib-0051] Mushonga, T. 2022. “Constitutional Environmental Rights and State Violence: Implications for Environmental Justice in Protected Forests.” Environmental Justice 16, no. 3: 194–202. 10.1089/env.2022.0059.

[nin12690-bib-0052] National Community‐Based Organization Network . 2004. What is a CBO? Ann Arbor, MI, USA: National Community‐Based Organization Network. https://sph.umich.edu/ncbon/about/whatis.html.

[nin12690-bib-0053] Nicholas, P. K. , and S. Breakey . 2017. “Climate Change, Climate Justice, and Environmental Health: Implications for the Nursing Profession.” Journal of Nursing Scholarship 49, no. 6: 606–616. 10.1111/jnu.12326. 28749596

[nin12690-bib-0054] Passarelli, D. , F. Denton , and A. Day . 2021. Beyond Opportunism: The UN Development System's Response to the Triple Planetary Crisis. New York, NY, USA: United Nations University.

[nin12690-bib-0055] Paynter, M. , K. Jefferies , L. Carrier , and L. Goshin . 2021. “Feminist Abolitionist Nursing.” Advances in Nursing Science 45, no. 1: 53–68. 10.1097/ANS.0000000000000385. PMC8757588 34148972

[nin12690-bib-0056] Perry, D. J. 2024. “Sharing the Space of the Creature: Intersubjectivity as a Lens Toward Mutual Human–Wildlife Dignity.” Nursing Inquiry 31, no. 1: e12587. 10.1111/nin.12587. 37533209

[nin12690-bib-0057] Polit, D. F. , and C. T. Beck . 2017a. “Data Collection in Qualitative Research.” In Nursing Research: Generating and Assessing Evidence for Nursing Practice, 10th ed., edited by D. F. Polit , and C. T. Beck , 506–529. Philadelphia, PA: Wolters Kluwer.

[nin12690-bib-0058] Polit, D. F. , and C. T. Beck . 2017b. “Sampling in Qualitative Research.” In Nursing Research: Generating and Assessing Evidence for Nursing Practice, 10th ed., edited by D. F. Polit , and C. T. Beck , 491–505. Philadelphia, PA: Wolters Kluwer.

[nin12690-bib-0059] Polivka, B. J. , R. V. Chaudry , and J. Mac Crawford . 2012. “Public Health Nurses' Knowledge and Attitudes Regarding Climate Change.” Environmental Health Perspectives 120, no. 3: 321–325. 10.1289/ehp.1104025. 22128069 PMC3295355

[nin12690-bib-0060] Ramsden, I. M. 2002. “Cultural Safety and Nursing Education in Aotearoa and Te Waipounamu.” Doctoral Dissertation, Victoria University of Wellington. https://www.croakey.org/wp-content/uploads/2017/08/RAMSDEN-I-Cultural-Safety_Full.pdf.

[nin12690-bib-0061] Redvers, N. , Y. Celidwen , C. Schultz , et al. 2022. “The Determinants of Planetary Health: An Indigenous Consensus Perspective.” Lancet Planetary Health 6: e156–e163. 10.1016/S2542-5196(21)00354-5. 35150624

[nin12690-bib-0062] Rockström, J. , J. Gupta , D. Qin , et al. 2023. “Safe and Just Earth System Boundaries.” Nature 619: 102–111. 10.1038/s41586-023-06083-8. 37258676 PMC10322705

[nin12690-bib-0063] Simon‐Ortiz, S. , S. Bilick , M. Frey , et al. 2024. “Community Power–Building Groups and Public Health Ngos: Reimagining Public Health Advocacy.” Health Affairs 43, no. 6: 798–804. 10.1377/hlthaff.2024.00035. 38830166

[nin12690-bib-0064] Stop Ecocide Foundation . 2021. *Independent expert panel for the legal definition of ecocide: Commentary and core text* (p. 12). https://static1.squarespace.com/static/5ca2608ab914493c64ef1f6d/t/60d7479cf8e7e5461534dd07/1624721314430/SE+Foundation+Commentary+and+core+text+revised+%281%29.pdf.

[nin12690-bib-0066] Sugla, R. 2021. “Again at the Altar on Climate Solutions and Colonialism.” In Required Reading: Climate Justice, Adaptation, and Investing in Indigenous Power, edited by NDN Collective Climate Justice Campaign , 101–118. Burnaby, BC, Canada: Hemlock Printers.

[nin12690-bib-0067] Thomas, N. A. , B. Owen , A. L. Ersig , and L. C. Bratzke . 2023. “Pathways and Processes to the Embodiment of Historical Trauma Secondary to Settler Colonialism.” Journal of Advanced Nursing 79, no. 11: 4218–4227. 10.1111/jan.15818. 37553851

[nin12690-bib-0068] Travers, J. L. , E. C. Schenk , W. E. Rosa , and P. K. Nicholas . 2019. “Climate Change, Climate Justice, and a Call for Action.” Nursing Economic$ 37, no. 1: 9–12. https://digitalcommons.providence.org/cgi/viewcontent.cgi?article=2311&context=publications.

[nin12690-bib-0069] United Nations Environment Programme . 2021. *Making Peace With Nature: A Scientific Blueprint to Tackle the Climate, Biodiversity and Pollution Emergencies* (p. 168). https://wedocs.unep.org/xmlui/bitstream/handle/20.500.11822/34948/MPN.pdf.

[nin12690-bib-0070] Wang, C. , and M. A. Burris . 1997. “Photovoice: Concept, Methodology, and Use for Participatory Needs Assessment.” Health Education & Behavior 24, no. 3: 369–387. 10.1177/109019819702400309. 9158980

[nin12690-bib-0071] Whyte, K. P. 2018. “Indigenous Science (Fiction) for the Anthropocene: Ancestral Dystopias and Fantasies of Climate Change Crises.” Environment and Planning E: Nature and Space 1, no. 1–2: 224–242. 10.1177/2514848618777621.

[nin12690-bib-0072] Whyte, K. P. 2021. “Towards and Indigenous Energy Transition: Justice, Renewables, and Infrastructure.” In Required Reading: Climate Justice, Adaptation, and Investing in Indigenous Power, edited by NDN Collective Climate Justice Campaign , 211–232. Burnaby, BC, Canada: Hemlock Printers.

[nin12690-bib-0073] Willis, D. G. , and D. M. Leone‐Sheehan . 2019. “Spiritual Knowing: Another Pattern of Knowing in the Discipline.” Advances in Nursing Science 42, no. 1: 58–68. 10.1097/ANS.0000000000000236. 30720514

[nin12690-bib-0074] World Health Organization (WHO) . 2021. Climate Change and Health. Geneva, Switzerland: World Health Organization. https://www.who.int/news-room/fact-sheets/detail/climate-change-and-health.

[nin12690-bib-0075] Zahner, S. J. 2005. “Local Public Health System Partnerships.” Public Health Reports® 120, no. 1: 76–83. 10.1177/003335490512000113. PMC1497678 15736335

[nin12690-bib-0076] Zust, B. , and R. Jost . 2022. “Public Health Awareness of Climate Change's Impact on Health.” Public Health Nursing 39, no. 4: 797–805. 10.1111/phn.13050. 35239215

