# Supplementary material for: Climate Justice Perspectives and Experiences of Nurses and Their Community Partners
Source: Nurs Inq. 2024 Dec 16;32(1):e12690. doi: 10.1111/nin.12690 (PMC11648356; doi:10.1111/nin.12690)
Supplement: Supplementary file 1 — Supporting information. [file NIN-32-e12690-s001.docx]

Supporting Information

Supporting Information 1. Climate injustice themes.

| **Theme** | **Definition** | **Subthemes** | **N**  **Excerpts** | **N**  **Photos** | **RN / CBO** |
| --- | --- | --- | --- | --- | --- |
| Severance from Nature | A colonial wounding from the severance of relationships within Nature. | - The colonial paradigm of disconnection from Nature - Desensitization to cultural violence - Numb to Indigenous suffering | 10 | 3 | RN: 1  CBO: 2 |
| Supremacy | Patterns of domination that disadvantage all but a few (LeClair, Luebke, et al., 2021). | - Colonialism - Environmental racism - Classism - Cis-hetero patriarchy - Speciesism | 30 | 6 | RN: 5  CBO: 4 |
| Corporate Capitalism | “Owners of the means of production are not expected to create products and hire laborers for the social good, but rather as a means of creating profit and accumulating further capital for their own self- interest” (p.4). (Kasser et al., 2007) | - Racial capitalism - Extraction of land and labor - Exploitation of low-income and marginalized populations - Corporate political power | 29 | 10 | RN: 5  CBO: 3 |
| Corporate Climate Pollution | Toxic waste from corporations who perpetuate | - Legacy pollution - Fenceline toxic   waste exposures | 50 | 8 | RN: 6  CBO: 5 |

| **Theme** | **Definition** | **Subthemes** | **N**  **Excerpts** | **N**  **Photos** | **RN / CBO** |
| --- | --- | --- | --- | --- | --- |
|  | climate change. | - Frontline climate change exposures - Knowingly pollutes - Creates sacrifice zones - Deflects responsibility. - Neglects occupational health - Threatens workers - Multispecies injustices |  |  |  |
| State Violence | The deliberate use of the institutions  of colonial state power to inflict violence, including anthropogenic climate disruption and removal of the right to access natural resources, thereby sustaining injustices around human dignity, culture, recognition, and the overall right to life. (Kojola & Pellow, 2021; Mushonga, 2022) | - Agency silos - Funding priorities - Inadequate laws, policies, and regulations - Neglects fenceline & frontline protections. - Partners with corporations to allow pollution - Partners with media to perpetuate harmful stories - Neglects to hold corporations accountable for ecocide / genocide - Surveillance of resident organizers - Silences own staff - Threatens or retaliates | 123 | 15 | All |

| **Theme** | **Definition** | **Subthemes** | **N**  **Excerpts** | **N**  **Photos** | **RN / CBO** |
| --- | --- | --- | --- | --- | --- |
|  |  | against resident organizers   - Fenceline community is silenced - Co-opts the EJ Movement - Makes sacrifice zones invisible - Worsens Social Determinants of Health - Prevents community healing - Lack of transparent communication about health risks - False sense of security |  |  |  |
| Mortality | The death rate or number of deaths in a population. (National Cancer Institute, n.d.) | - Death from cancers - Death from mine shafts - Multispecies - Intergenerational | 16 | 4 | RN: 2  CBO: 3 |
| Morbidity | Having a disease or a symptom of disease, or the amount of disease within a population. (National Cancer Institute, n.d.) | - Cancers - Respiratory diseases - Bone diseases - Heat-related illnesses - Neurological diseases - Multispecies - Intergenerational - Community lack of   knowledge of health risks | 25 | 5 | RN: 6  CBO: 3 |

| **Theme** | **Definition** | **Subthemes** | **N**  **Excerpts** | **N**  **Photos** | **RN / CBO** |
| --- | --- | --- | --- | --- | --- |
| Despair | The loss of hope. | - Fear of the unknown - Loss of work due to climate impacts - Sense of powerlessness - Dissociation to ongoing toxic exposures - Indigenous loss of spiritual connection within Nature - Secondary traumatic stress for RNs outside of the community | 11 | 4 | RN: 4  CBO: 4 |

Supporting Information 2. The transition from injustice to justice theme.

| **Theme** | **Definition** | **Subthemes** | **N**  **Excerpts** | **N**  **Photos** | **CBO / PHN** |
| --- | --- | --- | --- | --- | --- |
| Long Struggle | The struggle over a long period of time to fight for climate justice. | - Navigating and remembering toxic legacies - Hard, messy work - Tiny force against big structures - Navigating personal health risks as a member of the affected community - Feelings of isolation in   this work | 25 | 4 | RN: 6  CBO: 3 |

Supporting Information 3. Climate justice themes.

| **Theme** | **Definition** | **Subthemes** | **N**  **Excerpts** | **N**  **Photos** | **CBO / PHN** |
| --- | --- | --- | --- | --- | --- |
| Spiritual Relationships within Nature | Experience of finding spiritual meanings and healing relationships within Nature. | - Finding spiritual meaning within Nature - Healing with more-than-human relatives - Healing settler colonialism - Paradigm shift that prioritizes Indigenous worldviews - Intergenerational - Indigenous resilience | 32 | 10 | RN: 4 CBO: 2 |
| Belonging | Sense of belonging in communities where people live, learn, work, play, and pray. | - Social capital - Multicultural - Intergenerational - Sense of place | 6 | 4 | RN: 3 PHN: 3 |
| Abundance | Communities have local and regenerative economies. | - Multicultural and multigenerational development - Restorative justice - Energy and food sovereignty | 11 | 7 | RN: 1 PHN: 1 |
| Protected Communities | Public health is protected and assured. | - Political power - Solidarity - Public health information is accessed. - Government assures corporate responsibility and accountability. - Rights of Nature | 15 | 3 | RN: 3 CBO: 3 |
| Communities of Care | Community members and  allies care for | - Community   members care for each other. | 13 | 7 | RN: 5 CBO:  4 |

| **Theme** | **Definition** | **Subthemes** | **N**  **Excerpts** | **N**  **Photos** | **CBO / PHN** |
| --- | --- | --- | --- | --- | --- |
|  | each other and Nature | - Community members care for Nature - Workers care for each other - Allyship and solidarity - Multicultural, multigenerational, and multispecies |  |  |  |
| Planetary Health and Well-being | Public health is inseparable from planetary health. | - Body-mind-spirit are interconnected within Nature. - Climate resilience - Healing historical trauma - Hope for future generations | 10 | 4 | RN: 3 CBO: 2 |
